# SNiPgenie: a tool for microbial SNP site detection from whole-genome sequencing data

**DOI:** 10.1099/acmi.0.001021.v3

**Published:** 2025-09-22

**Authors:** Damien Farrell, Viktor Perets, Stephen V. Gordon

**Affiliations:** 1 UCD School of Veterinary Medicine, University College Dublin, Belfield, Dublin 4, Ireland; 2 One Health Centre, University College Dublin, Dublin 4, Ireland; 3 UCD Centre for Experimental Pathogen Host Research, University College Dublin, Dublin 4, Ireland

**Keywords:** *Mycobacterium bovis*, SNP calling, software

## Abstract

Whole-genome sequencing (WGS) of microbial pathogens provides a high-resolution approach to antibiotic resistance profiling, lineage classification and outbreak surveillance. Identification of SNPs across the genome by alignment against a reference genome is the highest precision method of delineating strains. SNiPgenie is a bioinformatics pipeline designed to perform the entire variant calling process across many samples simultaneously. It was developed in the context of developing WGS tools to support the tracking of infection transmission of *Mycobacterium bovis* in livestock and wildlife, the principal causative agent of bovine tuberculosis in these populations. SNiPgenie may, however, be applied to other bacteria where evolutionary change can be tracked accurately using SNPs. The tool comes with both a command line and a user-friendly graphical interface. It can run on standard desktop or laptop computers. SNiPgenie and its documentation are available at https://github.com/dmnfarrell/snipgenie.

## Data Summary

The software and documentation are available at https://github.com/dmnfarrell/snipgenie under an open-source (GPL v3) licence. The source code for the current version (0.7.0) is archived on Zenodo at https://doi.org/10.5281/zenodo.14721564.

## Introduction

The introduction of whole-genome sequencing (WGS) has transformed the analysis of microbial populations, providing a genomic resolution far superior to earlier molecular typing methods [1]. WGS is now routinely used for comparative genomics with application to epidemiological studies and outbreak investigation. *Mycobacterium tuberculosis* complex (MTBC) species are especially suited for approaches whereby reads are aligned to a reference genome and genomic variants identified. The variants of interest are usually SNPs which provide a robust and stable signal of evolutionary change. Detected variants undergo filtering based on thresholds for factors such as presence in repeat regions, mapping quality or read depth. Typically, many samples are treated together. The SNPs for each sample are combined into sequence alignments, which are then used to construct phylogenetic trees or other measures of genetic relatedness.

The computational sequence of steps can be done separately, but it is much more convenient to create a so-called ‘pipeline’ that runs the commands in sequence. This term can describe anything from a single-use hard-coded script to reusable workflows that carry out additional tasks like file preparation. The term workflow is used in the following as a general description of any such tool. Various workflows exist for variant calling in studies with *M. tuberculosi*s [2] and are now well established and reliable. Leveraging of WGS in the study of bovine tuberculosis outbreaks in wildlife and cattle caused by *Mycobacterium bovis* is also increasing [3,5], with a growing number of tools specific to the purpose. Many existing tools are either command-line only and require varying degrees of computational expertise to install and run. Others are web-based like Galaxy [6], which may be suitable for beginners if a local instance is set up for them or for those willing to do their analysis online. Finally, there are commercial platforms like CLC Genomics Workbench [7] that provide graphical interfaces but are very costly and often closed-source, limiting flexibility and transparency. SNiPgenie attempts to fill this gap by offering a fully open-source workflow that includes both command-line and graphical user interface options. This makes it particularly accessible to users without specialist bioinformatics training.

SNiPgenie is a tool for microbial variant calling and phylogenetic analysis from raw read data. Most recently, we applied it in a comparative analysis of *M. bovis* and *M. tuberculosis* isolates with previously identified MTBC lineages in Ethiopia [8] and our recent as-yet-unpublished *M. bovis* survey of Ireland [9]. However, the tool will work with genomes from other clonal bacterial organisms where evolutionary relationships are tree-like and variant patterns are cleanly inherited. This tool has also been shown to work on an arbitrary haploid reference sequence, where it was used for assessing clone-specific SNPs and indels in single-copy human artificial chromosomes [10]. In this article, we detail the functionality and advantages of the tool, which should be useful for a non-specialist audience.

## Methods and results

**Fig. 1.**
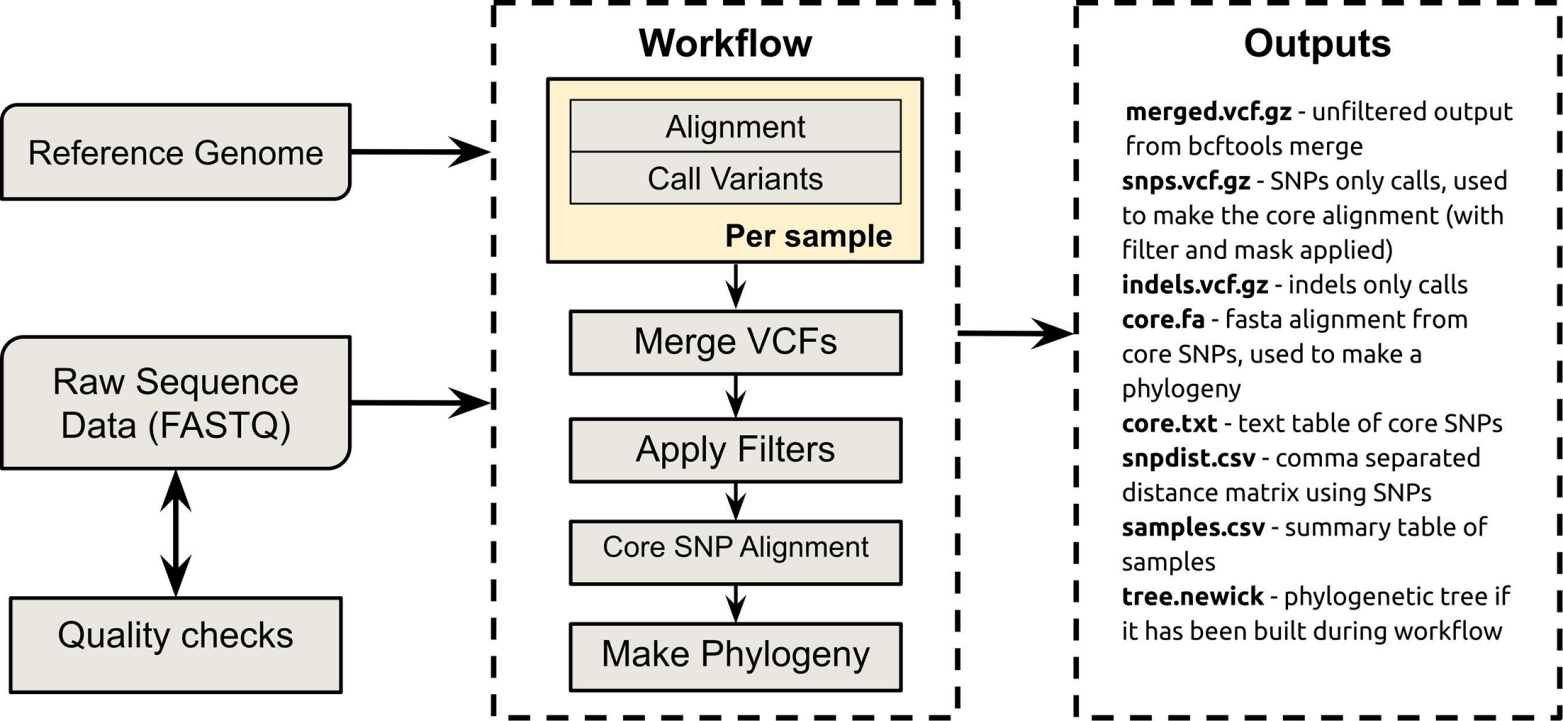
Workflow summary and outputs. Alignments and pileup are run per sample. The results are merged into one VCF, and the filters are applied to this. Then, a core alignment is generated from all variant positions.

The SNiPgenie tool was developed entirely in Python. The graphical user interface (GUI) is made using the Qt toolkit with PyQt5. It was developed on Ubuntu Linux but also runs on Windows via Windows Subsystem for Linux. The overall workflow is illustrated in Fig. 1. Alignment is done using BWA-MEM [11] (version 0.7.17). At the core of all the variant calling steps are samtools and bcftools (both htslib version 1.13) [12]. Version numbers are given as those used to test the software. There is a Python API that allows the workflow to be run directly with Python code (for example, this would facilitate re-use inside another program). All three methods produce the same output files. Runs can be resumed if stopped midway, depending on how far the workflow has progressed. For example, if not all files have been aligned, the run will start again without overwriting any completed files, unless requested. If all steps were previously completed and then re-run, output files will not be overwritten by default. The ‘overwrite’ flag is used to alter this behaviour. If users simply want to repeat the variant calling step without re-aligning, they can simply delete everything in the output folder and re-run. Command line usage is detailed below. Note that reads are not trimmed by this tool since trimming usually adds relatively little value to an SNP-calling pipeline [13]. Users may trim their own FASTQ files before input, if required.

### Benchmarking and performance

In a previous study [14], we benchmarked SNiPgenie against three other publicly available SNP-calling pipelines – vSNP [15], BovTB and MTBseq [16] – using simulated *M. bovis* WGS data derived from a high-prevalence bovine TB setting in Ireland. SNiPgenie demonstrated high performance, achieving recall and precision rates exceeding 99% when appropriate masking of problematic genomic regions (e.g. PE/PPE genes and mobile elements) was applied. The phylogenetic trees it produced closely matched the simulated ground truth and showed strong consistency with other pipelines when filtering was standardized. These findings highlight SNiPgenie’s reliability for high-resolution genomic epidemiology of bovine TB and underscore the importance of harmonized filtering strategies for cross-pipeline comparability. In the following sections, we provide some additional benchmarking in the light of recent updates to the SNiPgenie methodology.

### Variant calling methods compared

**Fig. 2.**
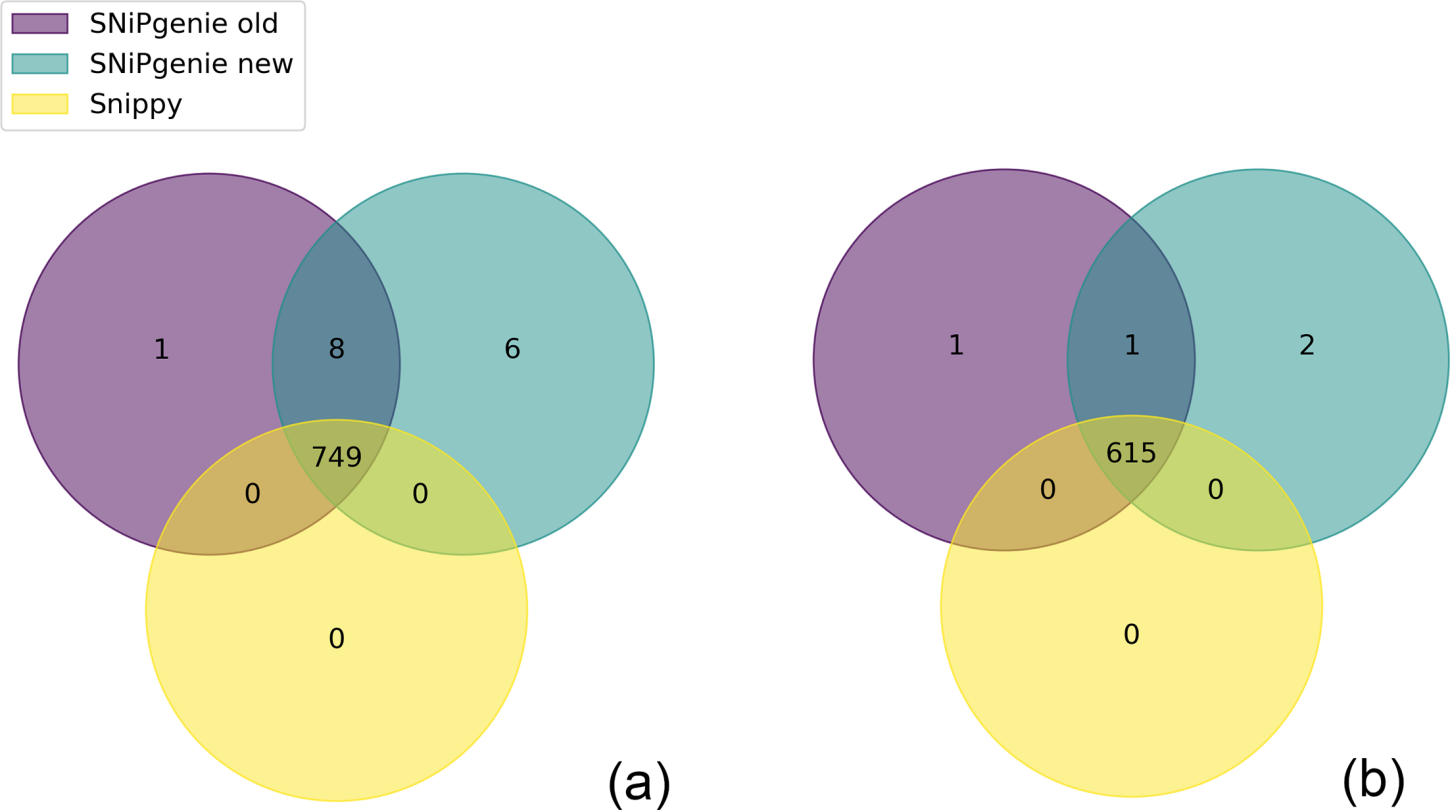
Venn diagram showing the agreement between the SNP positions identified in simulated *M. bovis* genomes by old and new variant calling methods. Snippy results are also included as an external comparison. (a) shows positions without masking, and (b) shows positions after the removal of the masked position.

The typical method of handling multiple files is to call variants individually for each aligned file, then merge the VCFs to get a file with all samples. The merged file can then be subject to further filtering. We also now follow this method using *bcftools mpileup*, *bcftools call* to extract SNPs and, finally, *bcftools merge* to combine all samples. However, at the time of our benchmarking paper, we used an approach that split the genome into segments and performed the pileup simultaneously for all samples together, then concatenated the VCFs into one file. This has since been replaced because it had the disadvantage of having to perform the pileup step when any new samples were added. To show that our previous benchmarks are still valid, we re-ran the old and new variant calling workflows on the same simulated *M. bovis* genomes. The results are presented in Fig. 2 which shows the overlap between core SNPs identified. We also ran Snippy [17] using no other filters except ‘--minqual 60’ on the same data. Fig. 2(a) shows the overlap for the three methods demonstrating some differences in old and new methods, with Snippy having no additional SNP positions. However, once masking of known regions is applied, the majority of these differences disappear, as shown in Fig. 2(b). This shows again the importance of masking problematic regions and that our previous results hold true for the new variant calling approach. The Jupyter notebook with code for these tests is available at https://github.com/dmnfarrell/snipgenie/blob/master/notebooks/benchmarking.ipynb.

### Requirements and speed

**Fig. 3.**
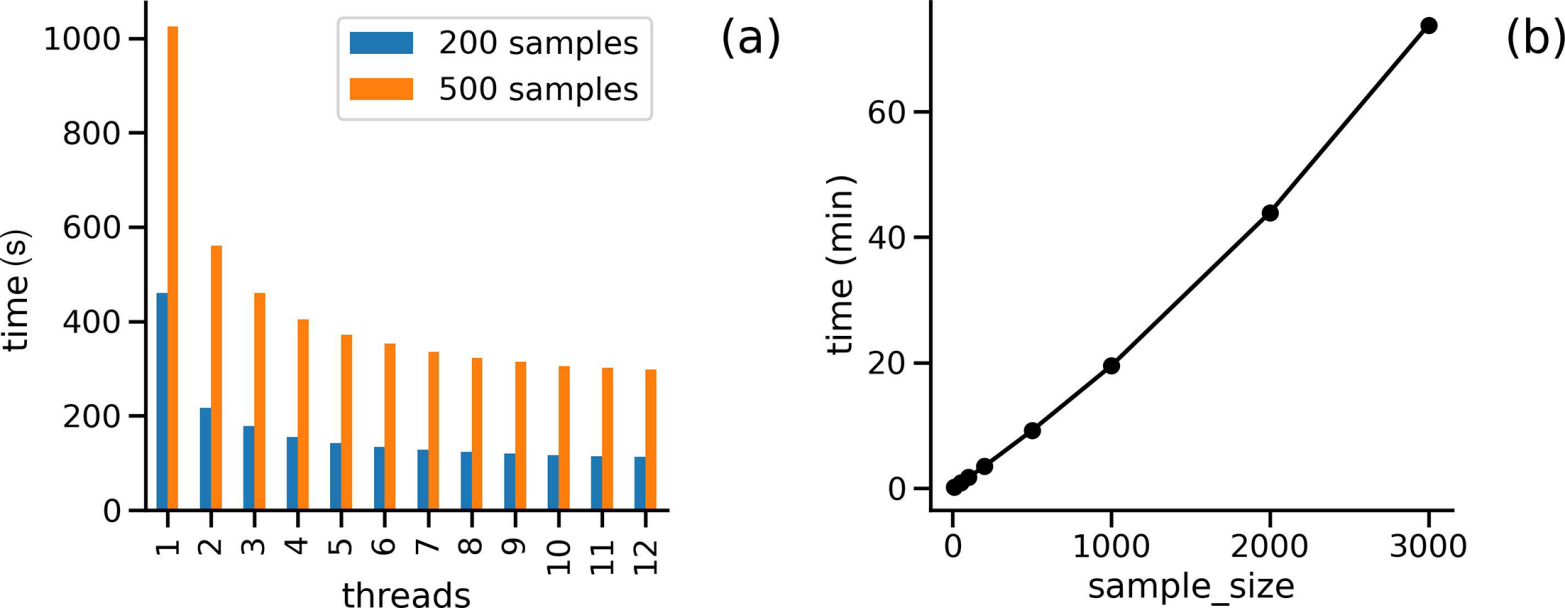
(a) Completion time for full SNiPgenie workflow plotted against the increasing number of threads, with 200 and 500 samples, respectively. (b) Workflow time as it increases with sample size. Tests were run on simulated SARS-CoV-2 genomes in both cases. Tree building was not included in the workflow in either case.

We have tested the speed in completion time for the full workflow using simulated SARS-CoV-2 sequences. These were created by simulating a phylogeny with TransPhylo [18] and using this to generate genome sequences using phastSim [19]. Artificial FASTQ files were then created for each genome with a custom Python function (available in SNiPgenie). Runs were tested on a desktop computer with a Ryzen 3900X processor and 32 GB of RAM. It should be noted that the majority of time is taken up by the alignment step, and we are, to some extent, measuring the speed of the aligner itself. Tree building was not included since this is optional and will not be relevant to some users. One of the most relevant factors for a modern computer is the ability to leverage multiple cores, so we explored this first. Fig. 3(a) shows the time taken versus the number of threads for 200 and 500 samples, respectively. It is clear that beyond four to eight threads, the gain in speed is minimal, consistent with the known multi-core performance of BWA-MEM [20]. The SARS-CoV-2 genome at ~30,000 bp is much smaller than an average bacterial genome, but the same pattern holds true. This also means that other pipelines will generally scale in the same way, since all rely on the same methodology. Fig. 3(b) that shows how compute time increases linearly with sample size, with 3,000 viral genomes taking just over 1 h to complete on our test desktop computer with 12 threads.

### Inputs

Files can be input from one or more directories with a variety of file naming formats. Folders are searched recursively for inputs with the extension *.f*q.gz. Each input (paired or single end) is given a unique sample name according to the file separator provided. Without unique names for each sample, the run will not be executed. If more customization is needed, specific sample and file locations may be specified in a manifest file. This is useful, for example, if you have duplicate file names in different folders so that unique sample names cannot be automatically parsed. There is no particular limit to the number of input samples. The workflow has been used in practice with 6,000 paired-end *M. bovis* sequences.

### Outputs

Output files, saved to the output folder, include the following: core.fa – fasta alignment from core SNPs, can be used to make a phylogeny; core.txt – text table of core SNPs; csq.tsv – consequence calls (if GenBank provided); csq_indels.tsv – consequence calls for indels; csq.matrix – matrix of consequence calls; snpdist.csv – comma-separated distance matrix using SNPs; samples.csv – summary table of samples.

### Selected options

Some of the options that can be specified at the command line or via the API are as follows:

Variant calling filter: may be provided as a string. The default is as follows:

“QUAL>=40 && FORMAT/DP>=30 && DP4>=4”

Mask file: SNP sites may be selectively masked from being included in the output. Genes encoding PE/PPE proteins in the * M. tuberculosis* complex, for example, are rich in repeat regions, especially GC-rich repetitive motifs. These repeats can cause issues during read alignment and are known to create false positive SNPs due to alignment artefacts caused by repeat regions. SNP callers may still attempt to call variants from these low-confidence regions. Therefore, such regions are typically excluded. The locations are provided in a bed file with the following columns: chromosome name, start and end coordinates of the regions. There are currently built-in mask files used for *M. bovis*, *M. tuberculosis* and *Mycobacterium avium* subsp. *paratuberculosis*, and if you select these genomes as references using the --species option, the mask will be used automatically.

Proximity filter: by default, a filter is run that excludes any variant positions within a given distance of each other. This is to prevent false positives resulting from alignment artefacts. This value can be set using the -pf option. The default value is 10; use 0 to ignore the filter.

Quality stats: by providing the --stats option, the mean depth and coverage of each sample will be calculated and saved to the samples.csv file. This can be used for quality control.

In the last step of the workflow, a maximum likelihood tree built from the SNP alignment (with RAxML [21] if installed) can be created. This is optional and selected with the -b or --buildtree option.

### Example command-line usage

The simplest usage is running with own reference fasta file and providing an input and output folder: snipgenie -r reference.fa -i data_files -o results

Use an in-built species genome as a reference. This will also supply an annotation and mask file specific to the species. The current options are Mbovis-AF212297, MTB-H37Rv, MAP-K10 and M.smegmatis-MC2155:

snipgenie -S Mbovis-AF212297 -i data_files -o results

Provide more than one folder:

snipgenie -r reference.fa -i data_files1 -i data_files2 -o results

Provide an annotation (GenBank format) for consequence calling:

snipgenie -r reference.fa -g reference.gb -i data_files -o results

Add your own filters and provide threads:

snipgenie -r reference.fa -i data_files -t 8 -o results` \

-f 'QUAL>=40 && INFO/DP>=20 && MQ>40'

### Use from Python

You can run the workflow from within Python by importing the ‘snipgenie’ package and invoking the WorkFlow class. You need to provide the options in a dictionary with the same keywords as the command line. Notice in this example, we are loading files from two folders.

import snipgenie

args = {'threads':8, 'outdir': 'results', 'labelsep':'-',

'input':['/my/folder/',

'/my/other/folder'],

'reference': 'sequence.fa', 'overwrite':False}

W = snipgenie.app.WorkFlow(**args)

W.setup()

W.run()

### Graphical interface

There is an optional desktop application that carries out the same workflow but provides an interactive environment that may be preferred by some users. It is installed along with the package and just requires a few additional dependencies, all available in Ubuntu. Basic usage is briefly described here. Once the package is installed, the program is invoked from the command line using ‘snipgenie-gui’. Before adding your FASTQ files, an output folder for results must be set. Samples can be loaded into the project from any folder by selection of files or using the ‘load folder’ method. Alternatively, a folder from a previous run with the command line tool can be loaded and the sample table automatically populated.

**Fig. 4.**
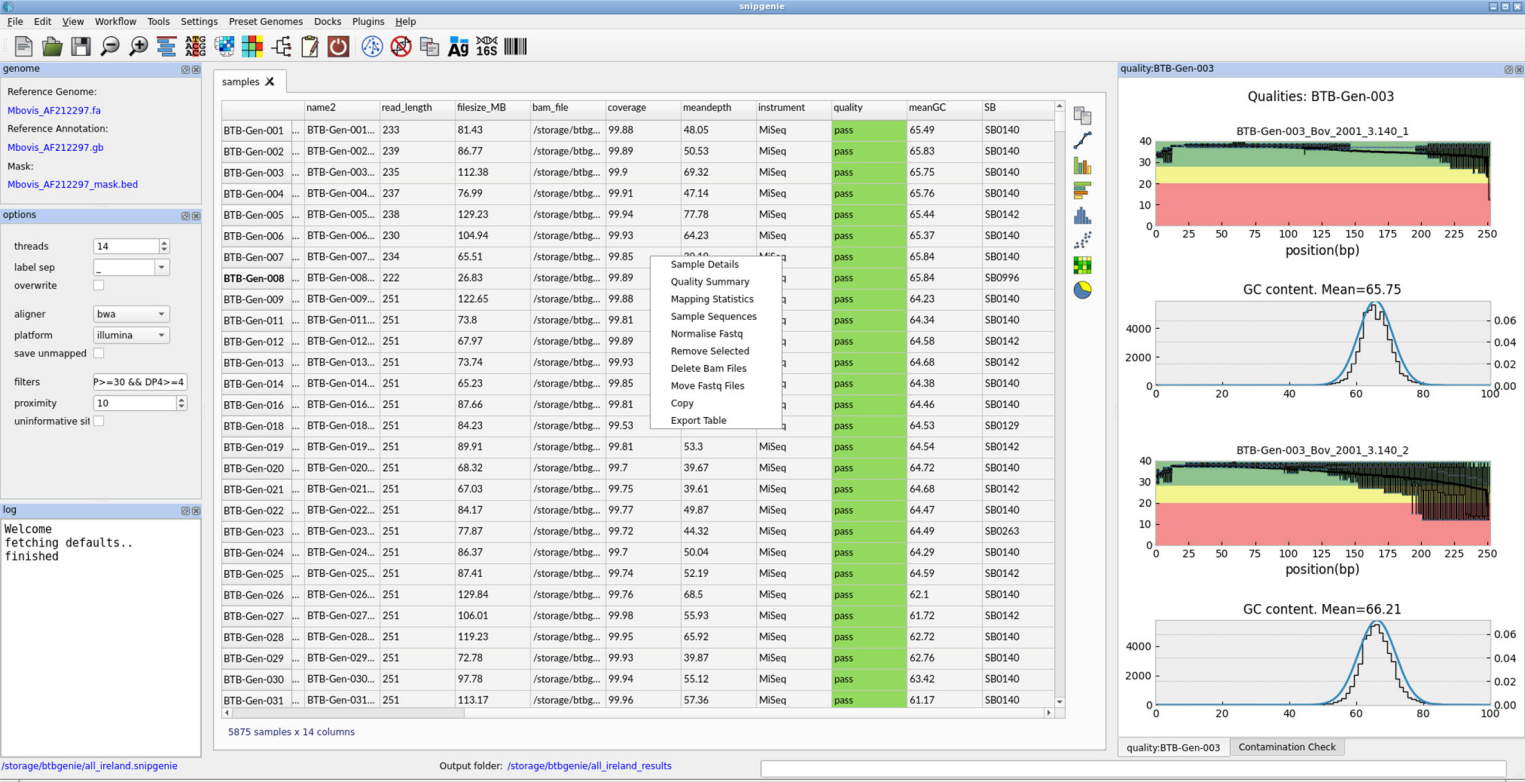
Graphical user interface layout. The interactive table in the centre contains all sample data showing the right-click menu. On the right are FASTQ quality plots. The left contains project settings. On the bottom left is a log/error window. These elements can be rearranged by dragging.

The interface layout is shown in Fig. 4. The main table is at the centre with sets of docked widgets at the left, right or bottom. Usually, plugins will appear on the right side when launched. Rows in the table can be selected to align or check the properties of individual samples. Mean depth, coverage, read length and mean GC can all be calculated. FASTQ quality for individual samples can be plotted in a manner similar to FastQC [22]. This will display (a) a plot of the mean read qualities vs. length and (b) a plot of GC distribution. Samples can be removed or added at any time. Simple graphical (bar, scatter or histogram) plots can be made from numerical data in the table for summary purposes, e.g. to plot per cent coverage across many samples. The table of SNPs derived in core.txt can be viewed interactively along with any VCF file in the result folder. The maximum likelihood tree built from the SNP alignment can be plotted. Finally, a table of metadata can be merged with the sample table. This can be used to colour the tree. Advanced users will probably want to create such visualizations themselves, but some of these functions will be helpful for beginners.

Extra functionality is available with several plugins which are presented in a docked pane next to the main table. Currently, there are plugins for the following tasks:

- Contamination check of reads against known genomes can be performed per sample
- Species-specific identification from reads using 16S databases. This requires you to assemble a genome which can be done inside the application using SPAdes [23]
- *M. bovis* spoligotype identification from reads

Further details are given in the documentation (https://github.com/dmnfarrell/snipgenie/wiki/).

## Discussion

Despite differences in design and implementation, the SNP-calling pipelines that we have previously evaluated – vSNP, SNiPgenie, BovTB and MTBseq – produced broadly similar results in terms of variant detection accuracy and phylogenetic reconstruction when standardized filtering was applied. All pipelines achieved high recall and precision, and their inferred phylogenies closely matched the simulated ground truth. Where pipelines do differ substantially is in flexibility, user interface and input requirements. These distinctions may not affect core analytical performance but can influence pipeline suitability depending on user expertise, analysis goals and integration into broader workflows. For example, BovTB outputs consensus genomes instead of core SNP alignments. Some pipelines, like MTBseq, include additional features such as resistance profiling and lineage classification. vSNP is relatively unique in that it allows visual inspection of SNPs in the form of formatted spreadsheets, though it appears to only handle inputs with each FASTQ pair in a separate folder, which is not very flexible. We found Snippy somewhat difficult to configure properly for multi-sample runs, though it did run efficiently when correctly used.

We would summarize the advantages of SNiPgenie as follows:

- a user-friendly graphical interface
- can be run directly in Python, so it could be used in another tool
- flexible input file/folder structure – any arbitrary folders can be used as input
- preset masks for certain bacterial species

Further development of the tool could include added plugins for the GUI. For example, a tool for users to create their own mask files.

## Conclusion

SNiPgenie provides a versatile and user-friendly solution for SNP detection and phylogenetic analysis, offering both command-line and graphical interfaces to cater to a diverse range of users. Its compatibility with multiple bacterial species, alongside some customizable options for variant calling, ensures adaptability to various research needs. By providing a graphical tool, SNiPgenie can help to bridge the gap between computational accessibility and advanced genomic analysis.

## Abbreviations

GUI: graphical user interface
MTBC: *Mycobacterium tuberculosis* complex
TB: bovine tuberculosis
WGS: whole-genome sequencing
